# Impact of oxygen deficiencies on magnetic properties of La_0.725_□_0.275_MnO_3−*δ*_ compounds

**DOI:** 10.1039/d5ra07059g

**Published:** 2026-04-17

**Authors:** W. Ben Jdidia, H. Gharsallah, M. Smari, M. Bejar, E. K. Hlil, E. Dhahri

**Affiliations:** a Laboratoire de Physique Appliquée, Faculté des Sciences, Université de Sfax Tunisia bejar_moez@yahoo.fr +216 74 676609 +216 98 333 873; b Institut Préparatoire aux Études d'Ingénieur, Université de Sfax Tunisia; c Faculté des Sciences, Université de Monastir Tunisia; d Institut Néel, CNRS Université J. Fourier BP166 38042 Grenoble France

## Abstract

In this study, oxygen-deficient La_0.725_□_0.275_MnO_3−*δ*_ (*δ* = 0.00, 0.15, 0.25 and 0.35) compounds were synthesized using the sol–gel method. The elemental composition was confirmed by energy-dispersive X-ray spectroscopy (EDX) measurements, while X-ray photoelectron spectroscopy (XPS) measurements were employed to quantify and validate the oxygen deficiency levels. X-ray diffraction (XRD) analysis revealed that all samples crystallize with a rhombohedral structure in the *R*3̄*c* space group and exhibit nanometric crystallite sizes. Magnetic measurements demonstrated that the Curie temperature (*T*_C_) and magnetization (*M*) are strongly dependent on the oxygen deficiency (*δ*). The field-cooled/zero-field-cooled (FC/ZFC) magnetization curves reveal a pronounced magnetic irreversibility in all compounds, which becomes more marked as the *δ* value increases. This behavior is closely related to the enhancement of the magnetic anisotropy (MA) with increasing *δ*. Furthermore, analysis of the inverse magnetic susceptibility (*χ*^−1^(*T*)) shows clear deviations from the Curie–Weiss law at temperatures above the magnetic transition. These deviations are clearly observed for the compounds with *δ* = 0.00 and 0.15, become significantly weaker for *δ* = 0.25, and completely disappear for *δ* = 0.35. This evolution indicates that the magnetic inhomogeneities responsible for the non-Curie–Weiss behavior are progressively suppressed as *δ* increases, concomitant with strengthening of the magnetic anisotropy. Meanwhile, hysteresis loop measurements revealed a difference between the theoretical and experimental magnetization saturation values for the samples with *δ* = 0.00, 0.15 and 0.35. This disparity was assigned to a significant antiferromagnetic (AFM) contribution and to magnetic disorder on the nanoparticle surface. In contrast, the good agreement between the theoretical and experimental magnetic saturation for the compound with *δ* = 0.25 was attributed to the predominance of double exchange (DE) interactions.

## Abstract 2

A discrepancy between the experimental and predicted magnetization saturation values was discovered from the hysteresis loops. An antiferromagnetic component for samples with *δ* = 0.15 and 0.35 is cited as an explanation for this disparity.

## Graphical abstract

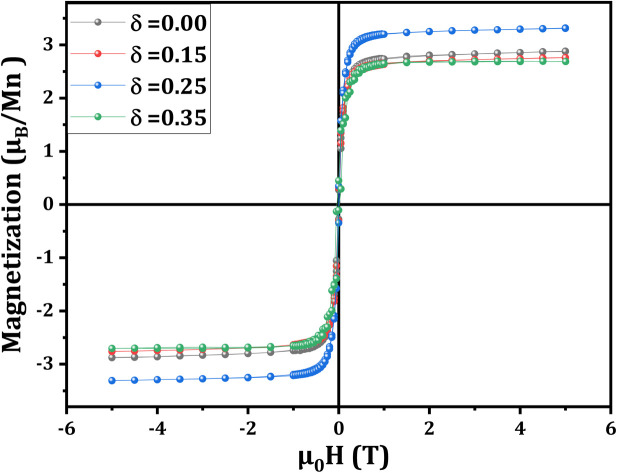

## 1. Introduction

Manganites with the general formula R_1−*x*_A_*x*_MnO_3_, where R is a rare-earth element such as lanthanum and A is an alkaline-earth element, have been a hot area of research, generating significant scientific interest and attracting considerable attention from researchers due to their wide range of technological applications.^1–6^ These materials are widely used in the domains of magnetic refrigeration^7–13^ and gas sensing.^14–16^ Their outstanding properties result from complex electronic interactions, which are strongly influenced by several structural and chemical parameters. Among the key factors, the preparation method and annealing temperature play a major role in optimizing the properties of manganites.^17–19^ In particular, their magnetic characteristics are directly related to the 
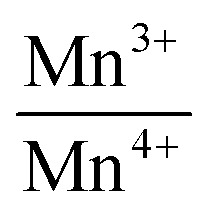
 ratio, which controls the DE mechanism. Multiple strategies have been proposed for adjusting this ratio, including substitution at the A-site with different elements.^20,21^

Beyond cationic substitution, other approaches have been explored, such as the creation of A-site vacancies.^22,23^ In this regard, the work of Dhahri *et al.*^24,25^ highlighted the introduction of oxygen deficiencies as another effective technique for modulating the magnetic properties of manganites.

Within this framework, these different methods equally demonstrate the existence of complex phenomena that influence the magnetic behavior of the materials. Among them, the SG behavior,^26^ the GP (ref. 27 and 28) and the AM (ref. 29) play a pivotal role. The emergence of these states is closely linked to several parameters, including the crystalline structure and crystallite size.^30^

Along this line, studies conducted by Mabrouki *et al.*^31^ focused on a series of manganites La_0.8_^3+^□_0.2_Mn_0.4+2*δ*_^3+^Mn_0.6−2*δ*_^4+^O_3−*δ*_^2−^ with crystallite sizes of about 25 nm. Their work corroborated the presence of a GP in one of the compounds, notably attributed to a structural distortion that leads to the emergence of a significant proportion of the orthorhombic phase. Because the amount of Mn^3+^ ions becomes equal to 100% at a rate of 0.3, the oxygen deficiency for this series cannot be greater than *δ* = 0.25.

To gain better and deeper insight into the impact of the formation of oxygen deficiencies *δ*, in this study, we chose to tackle the structural and magnetic properties of oxygen-deficient compounds La_0.725_□_0.275_MnO_3_. Increasing the A-site vacancy rate (0.275) can promote the creation of oxygen defects, leading to a value of *δ* = 0.35.

## 2. Sample preparation and characterization

### 2.1. Synthesis and novelty of the approach

While the controlled creation of oxygen vacancies in La_1−*x*_Sr_*x*_MnO_3−*δ*_ compounds using titanium metal as an oxygen getter is a well-established method developed in our laboratory, the present study introduces significant advancements. These advancements concern both the synthesis of the parent compound and the extent of the oxygen deficiency explored. The primary novelty lies in the use of the sol–gel method to synthesize the initial La_0.725_□_0.275_MnO_3_ powder, as opposed to the conventional solid–state reaction employed by Abdelmoula *et al.*^32^ This wet-chemical route ensures superior chemical homogeneity, a finer particle size, and higher reactivity of the precursor powder. Consequently, this leads to more uniform and controlled incorporation of oxygen vacancies during the subsequent reduction step. Furthermore, we extend the investigation to a much wider range of oxygen deficiencies, up to *δ* = 0.35, compared to the previously studied limit of *δ* ≤ 0.15. This allows us to probe new physical regimes and property modifications in this colossal magnetoresistance material.

### 2.2. Sol–gel synthesis of the parent compound

The starting compound La_0.725_□_0.275_MnO_3_ was synthesized using the sol–gel technique. The nitrate precursors La(NO_3_)_3_·6H_2_O and Mn(NO_3_)_2_·4H_2_O were dissolved in distilled water in stoichiometric quantities (eqn (1)); ethylene glycol and citric acid were added to ensure solution homogeneity.

1 (0.725)La(NO_3_)_3_·6H_2_O + Mn(NO_3_)_2_·4H_2_O → La_0.725_□_0.275_MnO_3_ + *Gaz*

The solution was slowly evaporated at 70 °C for 2 h in order to remove excess water and then heated to 180 °C to from a viscous gel. This gel was dried at 350 °C for 5 h to yield a solid precursor. The obtained powder was thoroughly ground and subjected to a stepwise calcination process at 450, 600 and finally 800 °C each for six hours to promote crystallization and remove organic residues.

### 2.3. Controlled creation of oxygen vacancies

The phase-pure La_0.725_□_0.275_MnO_3_ powder, as confirmed by XRD, was then used to prepare oxygen-deficient samples, La_0.725_□_0.275_MnO_3−*δ*_. This was achieved using the titanium getter method, following a protocol adapted from our previous work. A precise amount of the powder was sealed under vacuum in a quartz ampoule together with titanium metal chips.

The amount of titanium was calculated to achieve target oxygen deficiency values of *δ* = 0.15, 0.25, and 0.35, according to the reaction:^32^2
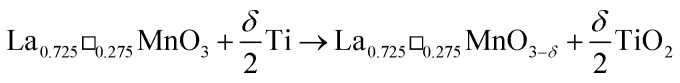


The sealed ampoule was heated at 600 °C for 13 days to ensure thermodynamic equilibrium and homogeneous oxygen diffusion throughout the sample volume. The actual oxygen deficiency, *δ*, was determined gravimetrically using a high-precision ultra-microbalance:^31^3
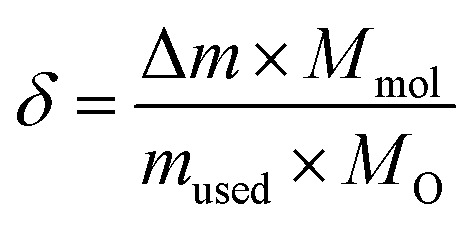
where Δ*m* denotes the mass difference before and after reduction; *M*_mol_ is the molar mass of the stoichiometric compound; *m*_used_ indicates the initial mass of the powder and *M*_O_ is the atomic mass of oxygen.

The measured *δ* values were in excellent agreement with the target values, with an error of 10^−4^, confirming the accuracy and reproducibility of the process.^31^

### 2.4. Structural, chemical, and magnetic characterization

The elemental composition and homogeneity of the samples were verified by energy dispersive X-ray spectroscopy (EDX). The chemical states and surface purity were investigated by X-ray photoelectron spectroscopy (XPS) using a monochromatic Al Kα source (1486.6 eV).

The crystal structure and phase purity of all samples (before and after reduction) were characterized at room temperature by X-ray diffraction (XRD) using a BRUKER D8 diffractometer equipped with a CuKα__1__ radiation source (*λ* = 1.5406 Å). Data were collected over the 2*θ* range of 25–90° with a step size of 0.02°.

The magnetic properties were investigated using a BS2 magnetometer at the Néel Institute in Grenoble, France. Magnetization measurements were performed as a function of the temperature and applied magnetic field up to 5 T.

## 3. Results and discussion

### 3.1. EDX study

**Fig. 1:**
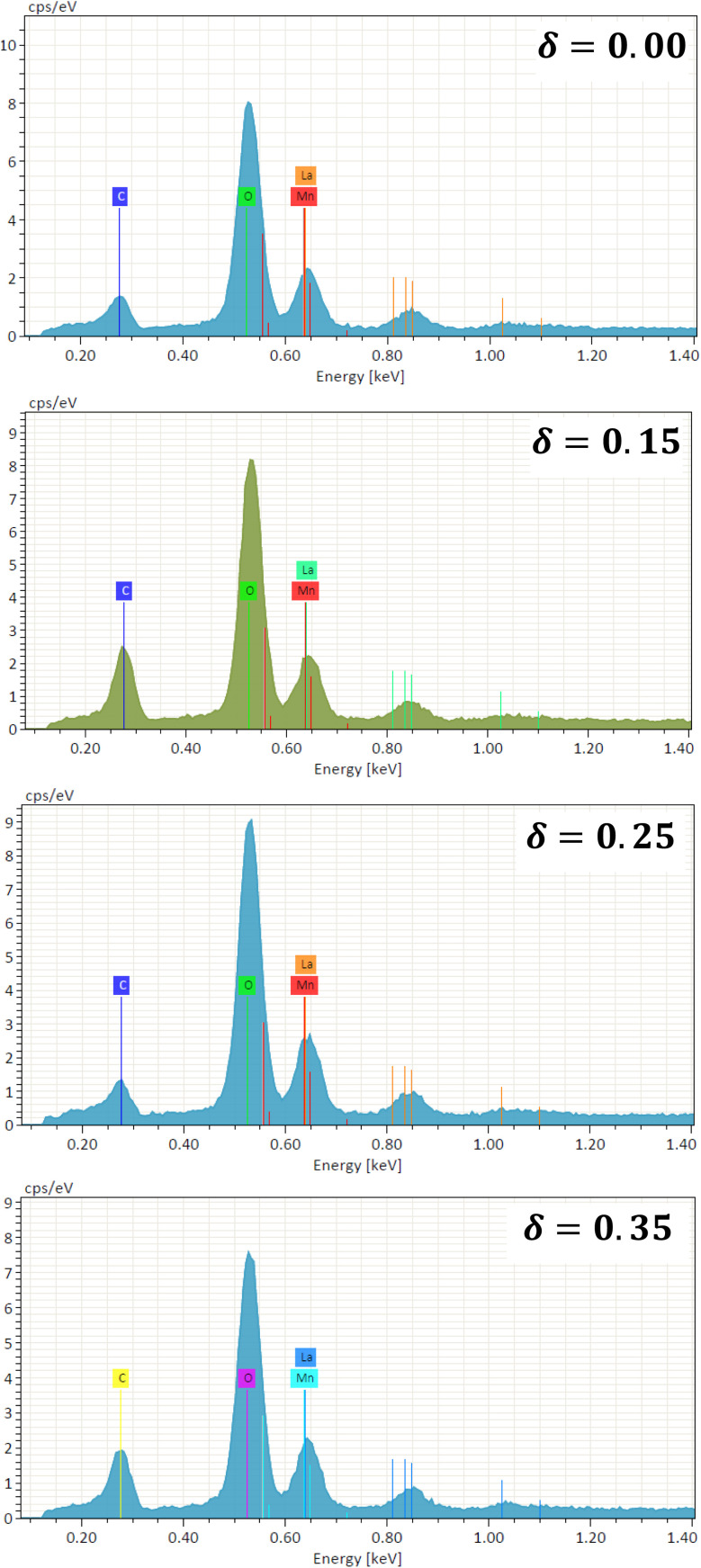
Typical scanning electron micrographs: EDX spectra of La_0.725_□_0.275_MnO_3−*δ*_ (*δ* = 0.00, 0.15, 0.25 and 0.35) compounds measured at room temperature.

To validate the elemental composition of the prepared compounds, we employed EDX. The spectra displayed in Fig. 1 revealed characteristic peaks corresponding to all constituent elements (La, Mn and O) of the La_0.725_□_0.275_MnO_3−*δ*_ compounds, confirming no significant elemental losses during the various preparation steps. The presence of carbon (C) in the sample spectrum can be attributed to the carbon tape used before the EDX analysis.

**Fig. 2:**
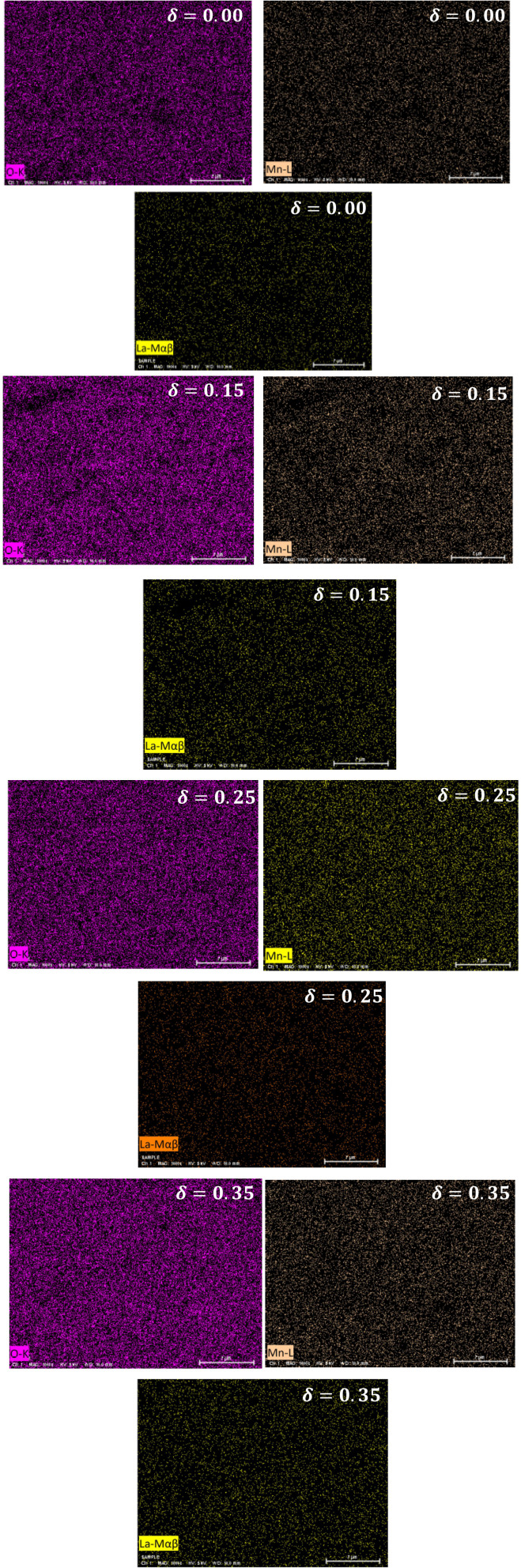
EDX mappings of O, Mn and La in La_0.725_□_0.275_MnO_3−*δ*_ (*δ* = 0.00, 0.15, 0.25 and 0.35) compounds at room temperature.

The measured atomic percentages, reported in Table 1, are close to the nominal values, which confirms the success of the preparation. This table reveals that the experimental La/Mn ratio values are very close to the theoretical ones. Additionally, EDX mapping demonstrates that the lanthanum (La), manganese (Mn), and oxygen (O) are uniformly distributed in the samples (Fig. 2).

**Table 1:** Atomic percentages of La_0.725_□_0.275_MnO_3−*δ*_ (*δ* = 0.00; 0.15; 0.25 and 0.35) compounds

*δ*		La (%)	Mn (%)	O (%)	La/Mn
0.00	Theoretical values	15.34395	21.16401	63.49204	0.72500
	Obtained values	15.45808	21.28083	63.26109	0.72639
	Relative error	0.74	0.55	0.36	0.19
0.15	Theoretical values	15.84703	21.85791	62.29506	0.72500
	Obtained values	15.78851	21.90552	62.30597	0.72075
	Relative error	0.37	0.22	0.02	0.59
0.25	Theoretical values	16.20115	22.34636	61.45249	0.72500
	Obtained values	16.05115	22.37335	61.57550	0.71742
	Relative error	0.93	0.12	0.20	1.05
0.35	Theoretical values	16.57146	22.85713	60.57141	0.72500
	Obtained values	16.36760	22.58966	61.04274	0.72456
	Relative error	1.23	1.17	0.78	0.66

### 3.2. XPS study

The XPS survey spectra of the La_0.725_□_0.275_MnO_3−*δ*_ (*δ* = 0.00, 0.15, 0.25 and 0.35) compounds were analyzed using Casa XPS software (version 2.3.25). The Shirley background was applied for all Mn 2p spectra to account for inelastic electron loss. Peak fitting was performed using a mixed Gaussian–Lorentzian (GL (30)) lineshape, and the full width at half maximum (FWHM) was constrained to remain within ±10% for all Mn oxidation states to ensure consistency and physical relevance.

**Fig. 3:**
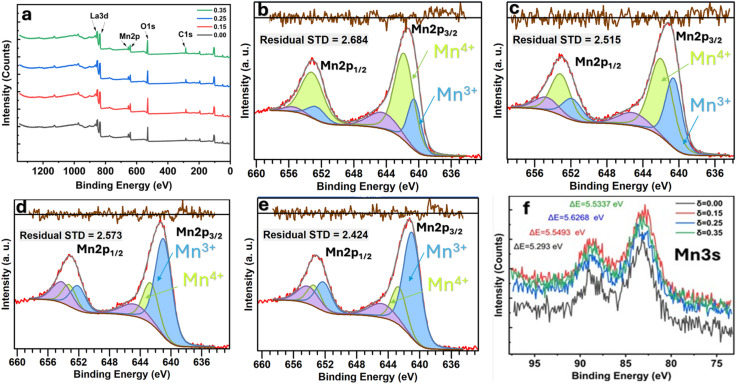
XPS profiles of La_0.725_□_0.275_MnO_3−*δ*_ (*δ* = 0.00, 0.15, 0.25 and 0.35) compounds: (a) survey, (b–e) Mn 2p deconvolution, and (f) Mn 2p multiplet splitting.

These spectra show only La, Mn, O, and adventitious C (Fig. 3a).^33^ There are no detectable contaminants. All fittings exhibit excellent agreement between the experimental (red) and fitted (black) spectra, with residual standard deviation (STD) values ranging from 2.42 to 2.68, indicating high-quality fits and consistent fitting parameters across all compounds. These findings validate the strength and reproducibility of the Mn 2p analysis, confirming the reliability of the extracted Mn^3+^/Mn^4+^ ratios addressed in the main text.

The C 1s peak at 284.8 eV was used as a reference for all of the spectra. We looked at the Mn core levels to figure out how the Mn^3+^/Mn^4+^ balance changed in the La_0.725_^3+^□_0.275_Mn_0.175+2*δ*_^3+^Mn_0.825−2*δ*_^4+^O_3−*δ*_^2−^ (*δ* = 0.00, 0.15, 0.25 and 0.35) compounds. For A-site-deficient perovskites La_0.725_□_0.275_MnO_3−*δ*_, charge neutrality requires *m*^3+^ + *m*^4+^ = 1, where *m*^3+^ = 0.175 + 2*δ* and *m*^4+^ = 0.825 − 2*δ* stand for the molar fraction of Mn^3+^ and Mn^4+^, respectively. High-resolution Mn 2p spectra (Fig. 3b−e) were modeled using Gaussian–Lorentzian lineshapes.^34^ The spin–orbit splitting of (11.6 ± 0.1) eV for all species and all compounds was fixed.^35^ The peaks were attributed to Mn^3+^ (2p_3/2_ ≈ 640.6 − 641.1 eV) and Mn^4+^ (2p_3/2_ ≈ 641.7 − 642.8 eV). Weak shake-up/charge-transfer satellites were added at a fixed offset (∼6 eV) as a constant fraction of the parent but were not included in the quantification.^36^ The full width at half maximum was restricted within the oxidation state and fixed across the series. The quantification ratios were determined from the main 2p_3/2_ components. The experimentally derived 
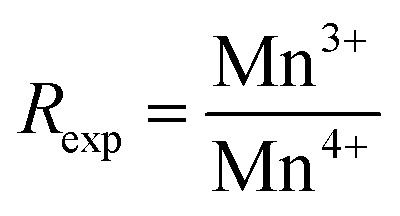
 ratios (Table 2) increased monotonically with *δ*: 0.22 (*δ* = 0.00), 0.79 (*δ* = 0.15), 1.78 (*δ* = 0.25), and 5.05 (*δ* = 0.35).^37^ The data as a whole adhered to the charge-balance trend, and there was a modest deficit in Mn^3+^ at the highest *δ*. Converting the ratios to bulk descriptors gave average Mn valences 
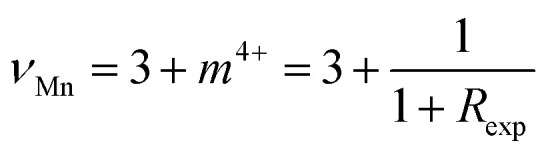
 of 3.82, 3.56, 3.36, and 3.17 for *δ* = 0.00, 0.15, 0.25, and 0.35, respectively.^38^ Using the inverse relation 
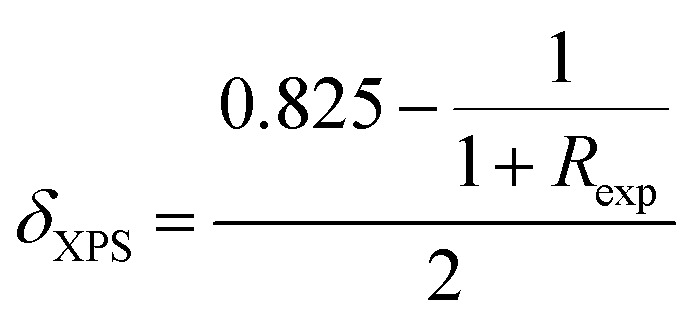
,^39^ we obtained *δ*_XPS_ ≈ 0.003, 0.133, 0.232 and 0.330, in excellent agreement with the nominal series. The Mn 3s spectra (Fig. 3f) were examined for multiplet splitting Δ*E*, which increased with increasing Mn reduction.^40^

**Table 2:** Mn 2p peak-fitting parameters and 
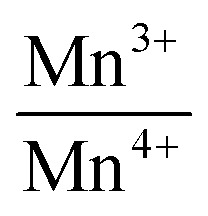
 experimental ratio for La_0.725_□_0.275_MnO_3−*δ*_ (*δ* = 0.00; 0.15; 0.25 and 0.35) compounds

*δ*	Mn2p_3/2_		
	Mn^4+^	Mn^3+^	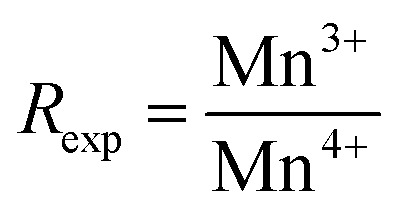
0.00	641.7246 eV	640.5665 eV	0.22
	47.99%	10.58%	
0.15	641.8508 eV	640.6083 eV	0.79
	33.05%	26.17%	
0.25	642.0356 eV	640.9182 eV	1.77
	23.12%	41.06%	
0.35	642.1317 eV	641.0571 eV	5.05
	10.25%	51.80%	

Mn 3s multiplet splitting (Δ*E*3s) was determined from the energy separation between both well-resolved 3s peaks (peak-to-peak distance) in the deconvoluted spectra. This separation originated from exchange coupling between the 3s core hole and 3d valence electrons. The measured Δ*E* values were directly taken from the experimental spectra after baseline correction, not from the fitted envelopes, ensuring minimal fitting bias. The experimental values of 5.293, 5.549, 5.627, and 5.534 eV at *δ* = 0.00, 0.15, 0.25, and 0.35 followed the Mn 2p-derived reduction (Mn^4+^ → Mn^3+^) and exhibited no characteristics of Mn^2+^.^41^ The slight non-monotonicity in the region between *δ* = 0.25 and 0.35 (≈0.09 eV) is within standard experimental scatter (charging/background selections) and does not affect the trend. This small non-monotonic variation can lie within the instrumental energy uncertainty (±0.1 eV) of the XPS setup. However, it may also reflect a minor contribution from local inhomogeneity or mixed-valence Mn^3+^/Mn^4+^ domains, which are typical of oxygen-deficient manganite. Such subtle effects are consistent with prior studies on La–Mn–O systems, exhibiting variable oxygen stoichiometry.^42–44^

Throughout the series, the Mn 2p binding energies vary subtly, trending to lower energies with increasing *δ*, and the relative Mn^3+^ peak area increases at the cost of that of Mn^4+^, both of which are clear signs of reduction caused by oxygen vacancies.^45^ The survey spectra verify the chemical purity, and the Mn 3s splittings independently verify the evolution of the Mn valence derived from Mn 2p fitting. Altogether, the findings confirm the controlled oxygen non-stoichiometry in La_0.725_□_0.275_MnO_3−*δ*_ and offer a quantitative connection between *δ*, the Mn^3+^/Mn^4+^ ratio, and the average Mn valence.

### 3.3. Structural study

**Fig. 4:**
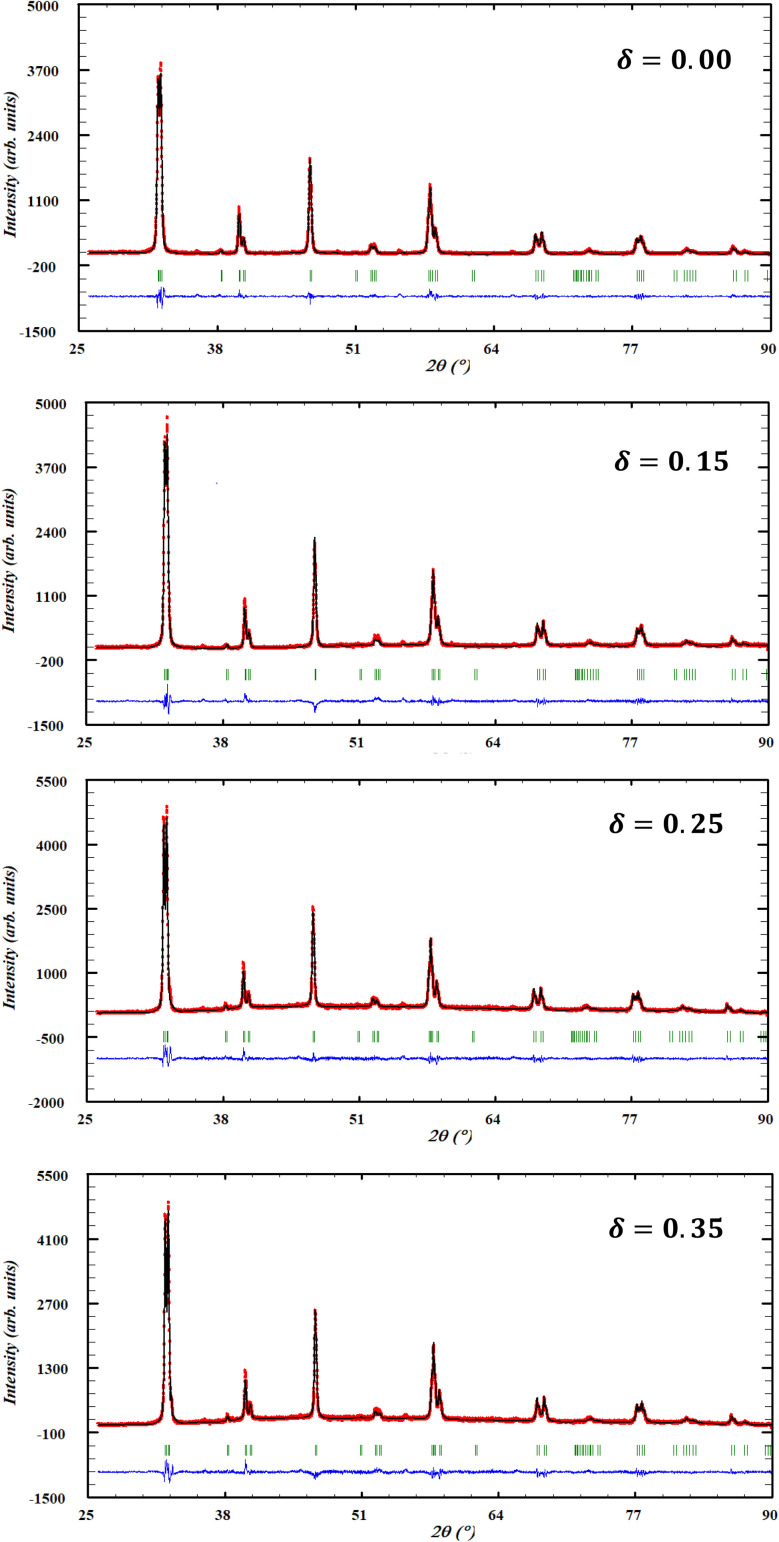
Observed (red) and calculated (black solid line) spectra and their difference patterns (blue) and the Bragg positions (green) of La_0.725_□_0.275_MnO_3−*δ*_ (*δ* = 0.00, 0.15, 0.25 and 0.35) compounds.

X-ray diffraction analysis revealed that all La_0.725_□_0.275_MnO_3−*δ*_ (*δ* = 0.00, 0.15, 0.25 and 0.35) compounds crystallize in the rhombohedral phase with *R*3̄c space group, as confirmed by Rietveld refinement using the FullProf program (Fig. 4). The space group assignment was further verified by comparison with the JCPDS database (card number 96-153-3630) with the assistance of Match 4 software.

The refinement results are displayed in Table 3. Accordingly, this table presents the crystallite sizes (*D*_SC_) determined from the XRD data using (eqn (4)):^46^4
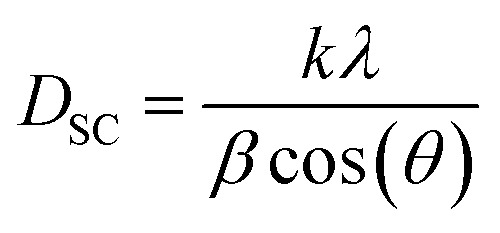
where *θ* refers to the diffraction angle of the most intense peak, *β* corresponds to the full width at half-maximum (FWHM) of this peak, *k* = 0.9 stands for the shape factor of the crystallites assumed to be spherical and *λ* denotes the wavelength of the radiation.

**Table 3:** Results of Rietveld refinement of XRD diffractograms of La_0.725_□_0.275_MnO_3−*δ*_ (*δ* = 0.00, 0.15, 0.25 and 0.35) compounds

*δ*	0.00	0.15	0.25	0.35
*a* (Å)	5.515(5)	5.509(7)	5.531(8)	5.536(2)
*c* (Å)	13.360(4)	13.353(2)	13.380(6)	13.388(0)
*V* (Å^3^)	58.664(6)	58.510(0)	59.101(8)	59.227(5)
*d*_Mn–O_ (Å)	1.963(1)	1.959(2)	1.971(4)	1.975(1)
〈Mn–O–Mn〉 (°)	163.539(5)	164.432(0)	162.200(0)	161.343(4)
*D*_SC_ (nm)	36.448(0)	38.337(0)	39.061(0)	39.796(2)
*W*/*W*_0_	0.093(3)	0.094(1)	0.091(9)	0.091(1)
*χ* ^2^	1.51	1.60	1.77	1.71

The crystallite size grows monotonically as the oxygen deficiency increases. The increase in the crystallite size is attributed to the increase in the average radius *r*_B_ of the B-site, determined using (eqn (5)):

5 *r*_B_ = (0.175 + 2*δ*)*r*_Mn_^3+^ + (0.825 − 2*δ*)*r*_Mn_^4+^

In fact, the creation of oxygen deficiencies in La_0.725_^3+^□_0.275_Mn_0.175+2*δ*_^3+^Mn_0.825−2*δ*_^4+^O_3−*δ*_^2−^ entails the partial reduction of Mn^4+^ ions (*r*_Mn_^4+^ = 0.53 Å) to Mn^3+^ ions, which have a larger ionic radius (*r*_Mn_^3+^ = 0.65 Å).

### 3.4. Magnetic study

**Fig. 5:**
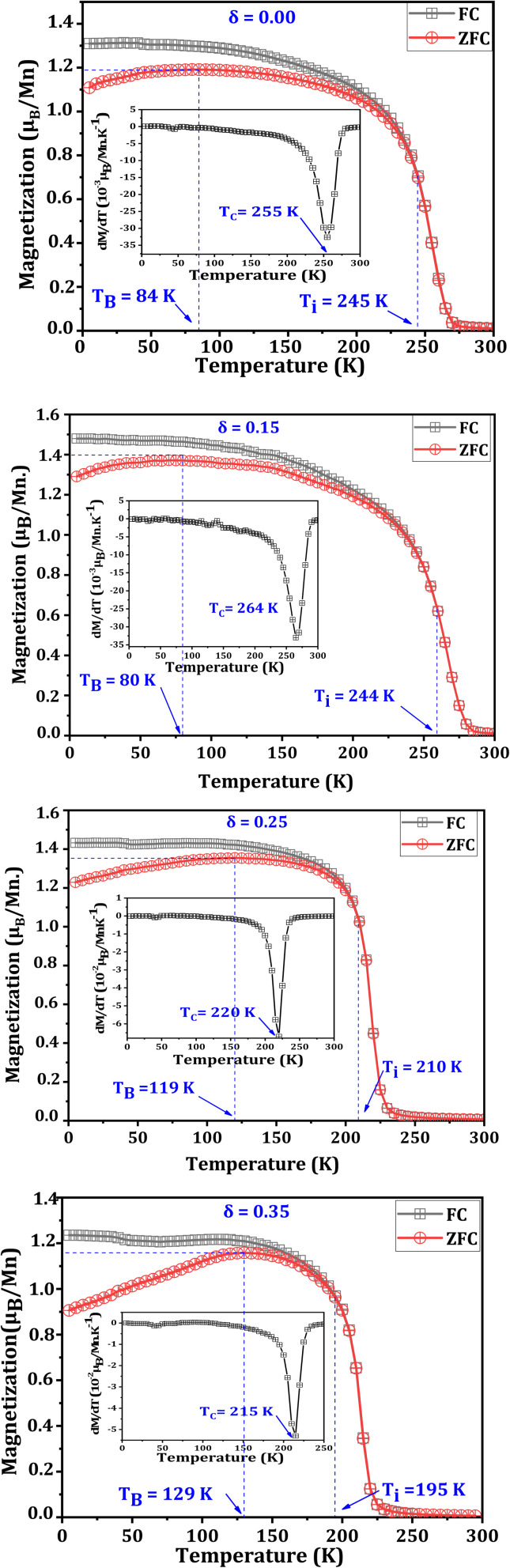
ZFC/FC magnetization *versus* temperature under an applied field of 0.05 T for La_0.725_□_0.275_MnO_3−*δ*_ (*δ* = 0.00, 0.15, 0.25 and 0.35) compounds. Insets: The variation of 
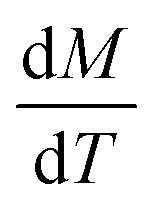
*versus* temperature.

Subsequently, we measured *M* as a function of temperature (*T*) in ZFC and FC modes, under an applied magnetic field of 0.05 T, for the La_0.725_^3+^□_0.275_Mn_0.175+2*δ*_^3+^Mn_0.825−2*δ*_^4+^O_3−*δ*_^2−^ (*δ* = 0.00, 0.15, 0.25 and 0.35) compounds (Fig. 5). The curves acquired in FC mode display a PM/FM transition, which is detected at the *T*_C_, with values of 255, 265, 220 and 215 K, for *δ* = 0.00, 0.15, 0.25 and 0.35, respectively. This temperature was determined from the d*M*/d*T* curves presented in the inset of Fig. 5. For the *δ* = 0.15 sample, we first observed a slight increase in the temperature following a slight improvement in the Mn–O–Mn angle and a reduction in the Mn–O distance, leading to an increase in the one-electron bandwidth (*W*) calculated using eqn (6)^31^ and presented in Table 3:6
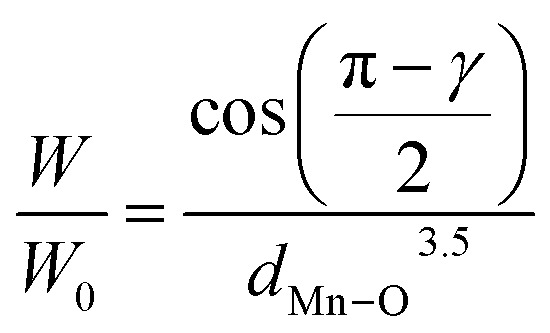
where *W*_0_ indicates a positive constant, *d*_Mn–O_ indicates the Mn–O distance and *γ* refers to the Mn–O–Mn angle.

Following the decrease in the Mn–O–Mn angle and the increase in the Mn–O distance, the decrease in *W* for the two other compounds (*δ* = 0.25 and *δ* = 0.35) is accompanied by a significant drop in *T*_C_.

However, it is inferred from Fig. 5 that the *M*(*T*) curves in the ZFC and FC modes have different characteristics that are affected by temperature. For temperatures above the irreversibility temperature (*T*_i_), both curves overlap, indicating reversible magnetic behavior. The *T*_i_ temperature corresponds to the point at which the FC and ZFC curves begin to diverge, marking a separation between the two measurement modes. It is observed that *T*_i_ decreases with increasing oxygen deficiency (Table 4).

**Table 4:** Blocking temperature (*T*_B_), irreversibility temperature (*T*_i_), Curie temperature (*T*_C_), anisotropy constant, anisotropy energy and Stoner–Wohlfarth field of La_0.725_□_0.275_MnO_3−*δ*_ (*δ* = 0.00, 0.15, 0.25 and 0.35) compounds

*δ*	0.00	0.15	0.25	0.35
*T*_B_ (K)	80	85	120	130
*T*_i_ (K)	245	248	210	195
*T*_C_ (K)	255	264	220	215
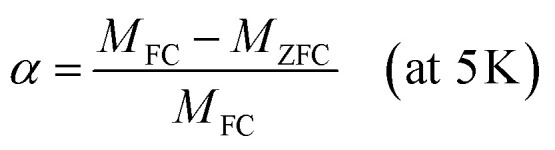	0.152	0.128	0.139	0.268
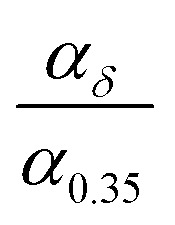	0.567	0.478	0.519	1
Anisotropy energy (KV′) (10^−20^ J)	2.845	3.023	4.269	4.628
*V*′ (10^7^ Å^3^)	2.535	2.950	3.120	3.300
Anisotropy constant (K) (10^6^ J m^−3^)	1.491	1.506	2.087	2.220
Stoner–Wohlfarth field *µ*_0_*H*_SW_ (T)	1.103	1.144	1.332	1.761

For the *T* > *T*_i_ region, *M* varies identically with temperature, whether during heating or cooling. This implies that the effect of the thermal cycle does not influence *M*.

At low temperatures (*T* < *T*_i_), the magnetic behavior becomes irreversible, indicating the existence of MA. Below this temperature, the spins freeze into a disordered state, generating irreversible SG/CG behavior, which is characteristic of materials exhibiting magnetic frustration as well as competing interactions.^47^ In numerous studies, researchers have identified divergence between the FC and ZFC modes as a result of this behavior.^48–50^ According to Pillai *et al.*,^51^ the ZFC curve indicates competition between the ferromagnetic (FM) and antiferromagnetic (AFM) interactions, which are of the DE and super-exchange types, respectively. AFM interactions are more prominent when the relative difference between the FC and ZFC curves is larger. This behavior can also be assigned to the presence of surface inhomogeneities related to spin disorder or MA, for compounds with nanometric crystallites sizes.^52^

This irreversibility between the FC and ZFC curves can be characterized by the relative difference between *M* in the FC and ZFC modes 
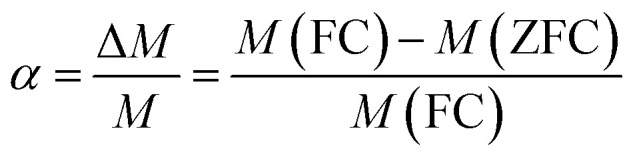
 at 5 K (Table 4). This deviation is 15.2%, 12.8%, 13.9% and 26.8%, respectively, for *δ* = 0.00, 0.15, 0.25 and 0.35. Based on the values of 
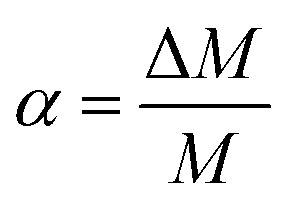
, we can assert that for compounds with *δ* = 0.00, 0.15 and 0.25, the divergence of the ZFC and FC curves remains relatively stable, suggesting a similar magnetic behavior within this composition range. For the compound with *δ* = 0.25, a notable increase of over 41% in the MA energy is detected. One might have expected an intensification of the SG/CG behavior, but this is not the case. Indeed, the SG/CG behavior results from competition between FM and AFM contributions. From this perspective, this observation can be attributed to strengthening of the DE interactions, which regulate the magnetic behavior in such a way that the FC/ZFC gap remains constant, suggesting weakening of the SG/CG behavior.

However, for the compound with *δ* = 0.35, a significant increase in this divergence is observed, indicating strengthening of the anisotropy and magnetic disorder effects.

These findings become clearer by comparing the coefficient 
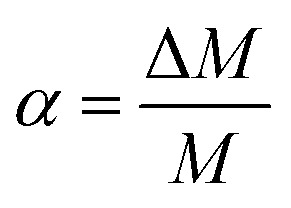
 for the different compounds with that of *δ* = 0.35. In fact, calculation of 
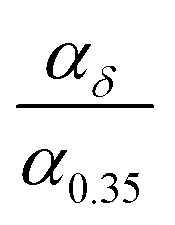
 (Table 4) leads us to a difference that does not exceed 57% for the compounds with *δ* = 0.00, 0.15 and 0.25, compared to that of *δ* = 0.35.

Through analyzing these curves, the blocking temperatures (*T*_B_) of the La_0.725_□_0.275_MnO_3−*δ*_ (*δ* = 0.00, 0.15, 0.25 and 0.35) compounds were determined and are outlined in Table 4. *T*_B_ is defined as the temperature below which the magnetic moments of a disordered system become frozen and can no longer follow thermal variations on experimental timescales.^53^ In this respect, for temperatures below *T*_B_, the magnetic anisotropy energy outweighs the thermal agitation energy. For such temperatures, the measurement time (*τ*_m_ = 155 s) is less than the Néel relaxation time *τ*_N_, which follows the Arrhenius law (eqn (7)). Thus, the magnetic moment is frozen, as indicated by the following equation:7
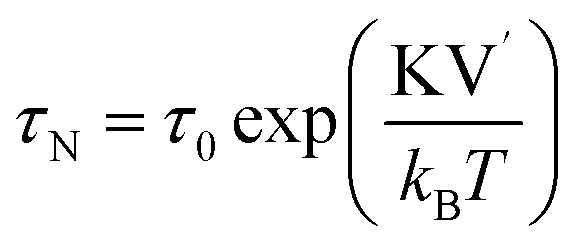
where *K* denotes the magnetic anisotropy constant, *k*_B_ refers to the Boltzmann constant, *τ*_0_ corresponds to the relaxation time for a zero-energy barrier, and *V*′ expresses the particle volume indicated by: 
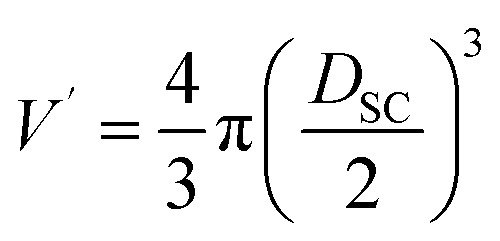
, where *D*_SC_ denotes the crystallite size.

Using the Arrhenius law (eqn (7)), when *τ*_N_ = *τ*_m_, *i*.*e*. for *T* = *T*_B_, the anisotropy energy (KV′)^47^ was determined. Knowing the values of the volume *V*′, we were able to determine the value of the magnetic anisotropy constant *K*. The values of KV′, *V*′ and *K* are summarized in Table 4.

The data in Table 4 suggest that a rise in the oxygen deficiency (*δ*) correlates with an increase in the crystallite size, which in turn leads to a larger particle volume, accompanied by a rise in the magnetic anisotropy constant *K* and consequently KV′ value.

Compared to the anisotropy energy obtained for the compound with *δ* = 0.00, the rise for the compound with *δ* = 0.15 is still rather small. For this reason, the FC/ZFC gap remains almost the same compared to that for *δ* = 0.00, indicating that the SG/CG behavior did not change at *δ* = 0.15. This stability has a direct consequence on the increase in the spontaneous magnetization compared to that for *δ* = 0.00. This improvement is ascribed to the increase in the proportion of Mn^3+^, thereby reinforcing the DE interactions in the case where *δ* = 0.15.

For the compound with *δ* = 0.25, a notable increase of over 41% in the magnetic anisotropy energy is detected. One might have expected an intensification of the SG/CG behavior, but this is not the case. Indeed, the SG/CG state results from competition between the FM and AFM contributions. From this perspective, this observation can be attributed to strengthening of the DE interactions, which regulate the magnetic behavior in such a way that the FC/ZFC gap remains constant, suggesting weakening of the SG/CG behavior. It is widely recognized that the DE reaches its highest efficiency when the Mn^3+^/Mn^4+^ ratio is approximately 7/3.^54,55^

In the case of the compound with *δ* = 0.35, the DE mechanism becomes much less important than in the case for *δ* = 0.25. This reduction in the DE interaction is accompanied by an increase in the magnetic anisotropy energy, resulting in stronger SG/CG behavior and subsequently a widening of the FC/ZFC gap.

This monotonic evolution of the magnetic anisotropy energy is confirmed by the evolution of the Stoner–Wohlfarth field, provided by eqn (8):^56^8
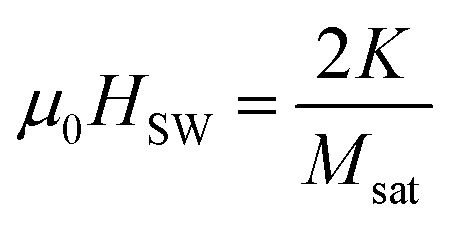
where, *K* indicates the uniaxial magnetic anisotropy constant (Table 4), *M*_sat_ refers to the experimental saturation magnetization (Fig. 7 and Table 5), *µ*_0_ denotes the vacuum permeability and *µ*_0_*H*_SW_ stands for the field required to reverse the magnetization in the direction of the easy magnetization axis. In other words, it corresponds to the field required to overcome the magnetic anisotropy.

**Table 5:** Theoretical and experimental values of magnetization saturation *M*_sat_ of La_0.725_□_0.275_MnO_3−*δ*_ (*δ* = 0.00, 0.15, 0.25 and 0.35) compounds

*δ*	Theoretical *M*^the^_sat_ (*µ*_B_/Mn)	Experimental *M*^exp^_sat_ (*µ*_B_/Mn)	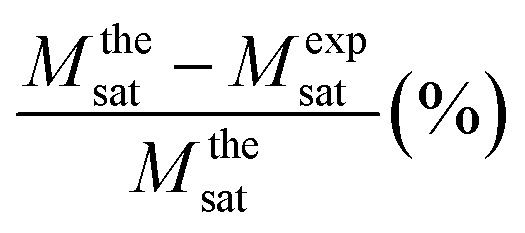
0.00	3.17	2.85	10
0.15	3.47	2.77	20
0.25	3.67	3.33	9
0.35	3.87	2.68	30

**Fig. 6:**
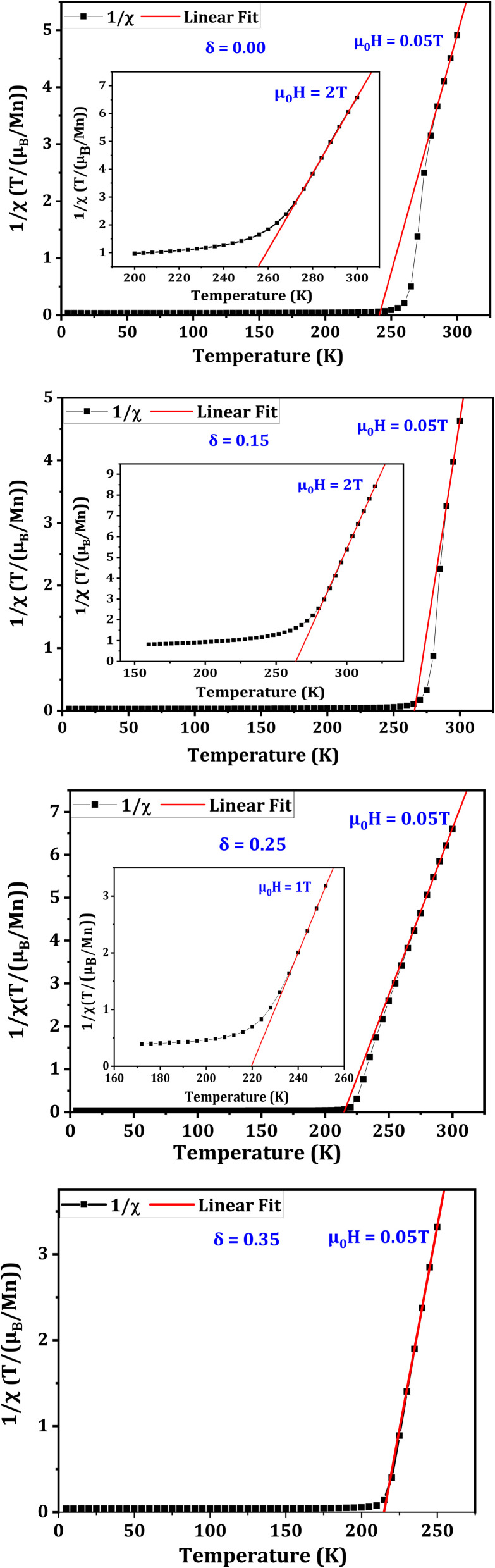
Temperature dependence of inverse susceptibility (*χ*^−1^) measured under an applied magnetic field (*µ*_0_*H*) of 0.05 T for La_0.725_□_0.275_MnO_3−*δ*_ (*δ* = 0.00, 0.15, 0.25 and 0.35) compounds. Insets: Curves recorded under (*δ* = 0.00)*µ*_0_*H* = 2 T, (*δ* = 0.15)*µ*_0_*H* = 2 T and (*δ* = 0.25)*µ*_0_*H* = 1 T showing that the deviation from the Curie–Weiss law is progressively suppressed, indicating the disappearance of the Griffiths phase.

Fig. 6 traces the evolution of (*χ*^−1^) as a function of temperature (*T*), under a magnetic field (*µ*_0_*H*) of 0.05 T. For the compound with *δ* = 0.00, the *χ*^−1^(*T*) curve deviates from the Curie–Weiss law above *T*_C_, suggesting the presence of the GP.^57–61^ Numerous studies have reported an analogous behavior, validating the existence of GP. The latter is marked by the presence of FM clusters integrated in the paramagnetic (PM) phase, as reported in multiple studies.^42,43^ To confirm this hypothesis, it is highly needed to analyze the behavior of the *χ*^−1^(*T*) curve under higher magnetic fields. As highlighted, for *δ* = 0.00, the application of a magnetic field (*µ*_0_*H*) of 2 T can suppress the curvature observed above *T*_C_.

For the compound with *δ* = 0.15, a similar behavior is observed. Indeed, the GP is present and can be suppressed by *µ*_0_*H* = 2 T. However, for the compound with *δ* = 0.25, the GP becomes weak and an applied magnetic field of about 1 T is sufficient to eliminate it. Although the anisotropy energy (KV′) is very high for this compound, it does not have such a significant impact on the SG/CG behavior or the GP. As a result, we can assert that the DE interactions in this compound substantially control the magnetic interactions. This keeps the lattice well organized and prevents the SG/CG state as well as GP from being properly established in the lattice. In contrast, for the *δ* = 0.35 compound, with a further increase in KV′, we notice that the SG/CG behavior becomes more important than in all other compounds, while GP becomes almost non-existent. This is confirmed through the shape of *χ*^−1^(*T*) curve, which becomes almost linear and follows the Curie Weiss law. Thus, it may be deduced that the SG/CG behavior is strengthened and GP is weakened as MA increases.

The structural, electronic, and magnetic characteristics of the La_0.725_□_0.275_MnO_3−*δ*_ series are closely interrelated, providing a coherent framework for understanding the observed magnetic behavior. XRD analysis reveals a rhombohedral lattice with subtle distortions that increase with increasing oxygen deficiency, while XPS measurements indicate a systematic variation in the Mn^3+^/Mn^4+^ ratio corresponding to *δ*. These lattice distortions modify the Mn–O–Mn bond angles and lengths, thereby tuning the DE interactions responsible for ferromagnetism. Simultaneously, the change in the Mn oxidation states alter the number of itinerant electrons, directly affecting the *T*_C_ and saturation magnetization. The combination of these structural and electronic inhomogeneities promotes magnetic frustration and anisotropy, which underpin the emergence of SG/CG behavior and influence the development of the GP. Although the *χ*^−1^(*T*) curves initially suggest a GP for *δ* = 0.00 and 0.15, this alone is insufficient evidence; the GP is supported by the presence of locally correlated FM clusters arising from lattice distortions and oxygen-induced electronic variations. Its progressive weakening with increasing *δ* correlates with the suppression of the GP, strengthening of the SG/CG behavior, and stronger XPS signals. Thus, the observed magnetic transitions reflect the intimate coupling between the lattice, charge, and spin degrees of freedom in oxygen-deficient manganites, demonstrating that structural and electronic changes jointly dictate the evolution of both glassy and cluster-like magnetic phenomena.

**Fig. 7:**
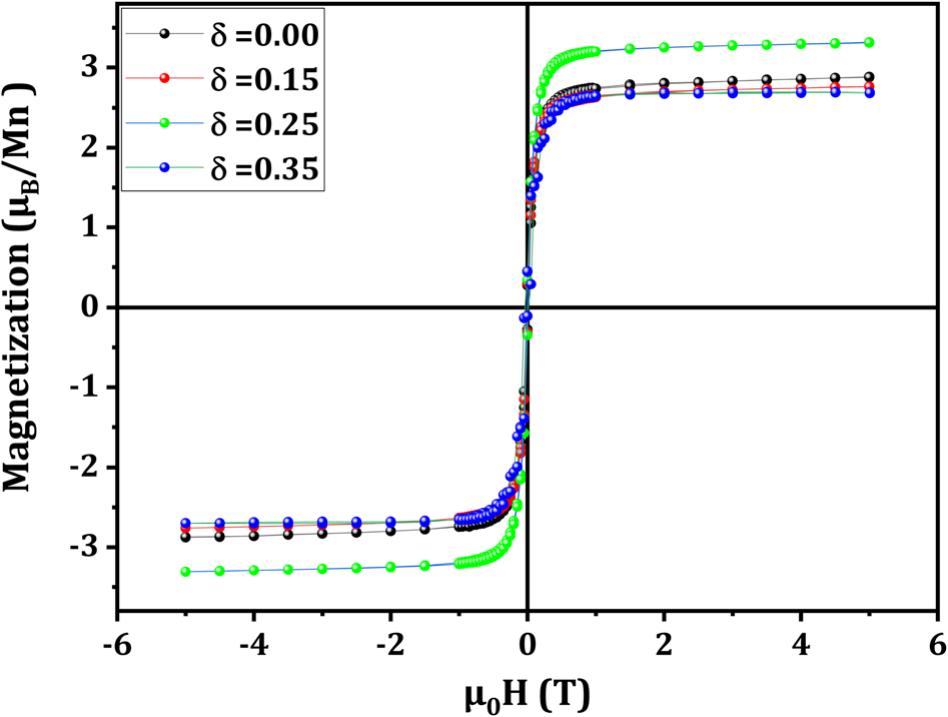
Applied magnetic field-dependence of the magnetization at 10 K for La_0.725_□_0.275_MnO_3−*δ*_ (*δ* = 0.00, 0.15, 0.25 and 0.35) compounds.

Hysteresis loops are a fundamental feature for characterizing the magnetic properties of materials, particularly saturation magnetization and the MA effect. Through analyzing these loops (Fig. 7), it is possible to assess the influence of magnetic interactions and structural effects on the alignment of moments under an external field.

In the current work, we compared the theoretical and experimental values of the magnetization saturation (*M*_sat_) of La_0.725_□_0.275_MnO_3−*δ*_ (*δ* = 0.00, 0.15, 0.25 and 0.35) compounds (Table 5).

The theoretical magnetization saturation value (*M*^the^_sat_) was determined using eqn (9):

9 *M*^the^_sat_ = (0.175 + 2*δ*) × *µ*_Mn_^3+^ + (0.825 − 2*δ*) × *µ*_Mn_^4+^

where *µ*_Mn_^3+^ = 4*µ*_B_ and *µ*_Mn_^4+^ = 3*µ*_B_ (ref. 31) represent the magnetic moments of Mn^3+^ and Mn^4+^ ions, respectively, expressed in *µ*_B_ (Bohr magnetons).

For the *δ* = 0.00 compound, the experimental and theoretical values of the saturation magnetization are equal to 2.85 and 3.17*µ*_B_/Mn, respectively (Table 5). We thus infer a relative difference between these two values equal to 10%. The MA cannot account for this difference since a *µ*_0_*H*_SW_ field of 1.103 T (Table 4) is able to overcome this anisotropy while the field reaches 5 T. It can be accordingly concluded that 10% of the magnetic moments do not contribute to the saturation magnetization, which can be explained by the core–shell model.^62^ In this regard, it is worth noting that this model applies in the case of compounds that have nanometric crystallites. In this model, the core is ferromagnetically ordered while the shell is either totally or partially disordered, *i*.*e*., the spins are in a state of paramagnetism or ferrimagnetism.^31^ Thus, the real experimental saturation magnetization (*M*^exp^_sat_) value can be obtained using the following relation:^47^

10 *M*^exp^_sat_ = *ρ*_shell_*M*_shell_ + (1 − *ρ*_shell_) *M*_core_

where *ρ*_shell_ = 10% stands for the shell magnetization fraction, *M*_shell_ and *M*_core_ refer to the contributions of the shell and the core to the magnetization, respectively.

Within the core, Mn^3+^ and Mn^4+^ ions are parallel to each other, and *M*_core_ is specified in terms of:

11 *M*_core_ = 0.175 × *µ*_Mn_^3+^ + 0.825 × *µ*_Mn_^4+^ = 3.17*µ*_B_/Mn

Departing from the experimental magnetic saturation *M*^exp^_sat_, we estimate the shell magnetization fraction as follows:12
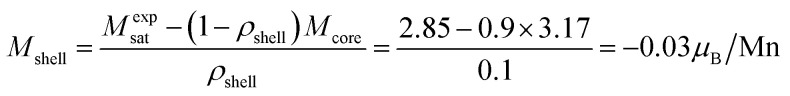


The gap between the experimental *M*^exp^_sat_ value and the theoretical *M*^the^_sat_ increases and reaches 20% for the compound with *δ* = 0.15. Since the crystallite sizes of both *δ* = 0.00 and *δ* = 0.15 compounds are nearly identical, the core–shell model cannot adequately account for this discrepancy, which is significantly larger than that for the *δ* = 0.00 compound. Similarly, with the *δ* = 0.00 compound, the MA cannot explain the extra 10% discrepancy from that found for the *δ* = 0.00 compound, since a *µ*_0_*H*_SW_ field of 1.144 T is able to exceed this anisotropy (Table 4), while the field reaches 5 T. Thus, this additional 10% gap can be assigned to the persistence of a robust AFM component within this compound.

For the *δ* = 0.25 compound, as pointed out previously, the DE interactions become dominant, implying that the FM order in this compound is very stable and no AFM order has a chance to establish itself. The magnetic field is significantly higher than the *µ*_0_*H*_SW_ field, which amounts to 1.332 T (Table 4). It is, therefore, able to align almost all the moments, which explains the good agreement between the theoretical and experimental values of *M*_sat_, with a relative difference equal to 9%. As stated for the case of the compound with *δ* = 0.00, this difference can be ascribed to the core–shell model.

For the *δ* = 0.35 compound, a high saturation magnetization was expected (*M*^the^_sat_ = 3.87*µ*_B_/Mn) (eqn (9)), but this is not the case. The discrepancy between the experimental and theoretical values is about 30%. The MA cannot explain this difference since the *µ*_0_H_SW_ field, which amounts to 1.761 T, is consistently smaller than the applied field (Table 4). Thus, this discrepancy can be attributed to the contribution of the core–shell model (of about 10%) as well as the robust AFM component (of about 20%).

## 4. Conclusion

The impact of oxygen deficiencies on the magnetic properties of oxygen-deficient La_0.725_□_0.275_MnO_3−*δ*_ (*δ* = 0.00, 0.15, 0.25 and 0.35) compounds was examined. Careful scrutiny was given to the compounds' compositions, structural properties, and magnetic properties. Energy dispersive X-ray spectroscopy (EDX) measurements confirmed the elemental composition of the compounds. XPS measurements corroborated that oxygen deficiency (*δ*) was created in the desired proportions. The determined 
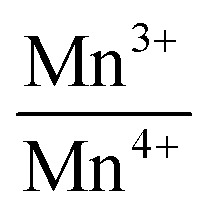
 ratio is very close to the theoretical ratio, confirming the successful preparation of the compounds. Structural analysis demonstrated that all compounds include crystallites with nanometric dimensions.

A deviation from Curie–Weiss's law in the compounds with *δ* = 0.00 and 0.15 was proven by inverse magnetic susceptibility *χ*^−1^(*T*) analysis. It was found that this behavior weakens for compounds with *δ* = 0.25 and vanishes for the compound with *δ* = 0.35. On the other hand, it was inferred that for the compound with *δ* = 0.25, the magnetic interactions were influenced by the double-exchange (DE) mechanism, which reached its maximum for this compound.

Ultimately, a discrepancy between the experimental and predicted magnetization saturation values was detected based on the hysteresis loops.

A substantial antiferromagnetic component, especially for the compounds with *δ* = 0.15 and 0.35, explains this disparity.

As a future perspective, an analysis of the progression of competition among the different magnetic interactions in the magnetic network as a function of the applied magnetic field can be conducted in subsequent studies.

## Conflicts of interest

The authors declare that they have no known competing financial interests or personal relationships that could have appeared to influence the work reported in this paper.

## Supplementary Material

RA-016-D5RA07059G-s001

## Data Availability

All data supporting this manuscript, including EDX mapping, X-ray diffraction data and magnetic measurement curves, are in the supplementary information (SI). Supplementary information is available. See DOI: https://doi.org/10.1039/d5ra07059g.
